# Anti-industry beliefs and attitudes mediate the effect of culturally tailored anti-smoking messages on quit intentions among sexual minority women

**DOI:** 10.1038/s41598-024-78207-7

**Published:** 2024-11-15

**Authors:** Brittany A Zulkiewicz, Jarvis T. Chen, Elaine P. Hanby, Shoba Ramanadhan, Juno Obedin-Maliver, Mitchell R. Lunn, N. F. N. Scout, Bob Gordon, Julia Applegate, Ana Machado, Kasisomayajula Viswanath, Jennifer Potter, Sixiao Liu, Andy S. L. Tan

**Affiliations:** 1 Annenberg School for Communication, University of Pennsylvania, Philadelphia, PA USA; 2 Department of Social and Behavioral Sciences, Harvard TH Chan School of Public Health, Boston, MA USA; 3 Harvard TH Chan School of Public Health, Boston, MA USA; 4 Department of Obstetrics and Gynecology, Stanford University School of Medicine, Stanford, CA USA; 5 The PRIDE Study/PRIDEnet, Stanford University School of Medicine, Stanford, CA USA; 6 Department of Epidemiology and Population Health, Stanford University School of Medicine, Stanford, CA USA; 7 Division of Nephrology, Department of Medicine, Stanford University School of Medicine, Stanford, CA USA; 8 National LGBT Cancer Network, Providence, RI USA; 9 California LGBT Tobacco Education Partnership, San Francisco, CA USA; 10 The Ohio State University, Columbus, OH USA; 11 CenterLink, Fort Lauderdale, FL USA; 12 McGraw-Patterson Center for Population Sciences, Dana-Farber Cancer Institute, Boston, MA USA; 13 The Fenway Institute, Boston, MA USA; 14 Beth Israel Lahey Health, Boston, MA USA; 15 Harvard Medical School, Boston, MA USA; 16 Department of Population Health Sciences, College of Medicine, University of Central Florida, Orlando, FL USA; 17 Abramson Cancer Center, Tobacco and Environmental Carcinogenesis Program, Philadelphia, PA USA; 18 Leonard Davis Institute of Health Economics, University of Pennsylvania, Philadelphia, PA USA

**Keywords:** Cessation, Tobacco industry, Disparities, Social marketing, Psychology, Risk factors

## Abstract

We conducted a longitudinal randomized controlled experiment between September 2021 and May 2022 to evaluate whether anti-tobacco industry beliefs and attitudes mediate the effect of culturally tailored anti-smoking messages on quit intentions among US young adult sexual minority women (SMW) ages 18–30 who smoke. Participants were randomized to view up to a total of 20 tailored versus non-tailored messages over one month. Outcomes were assessed at baseline and one-month follow-up. We fit a structural equation model testing the effect of LGBTQ + community-tailored, anti-smoking messages on quit intentions and mediating roles of anti-industry attitudes and beliefs (*n* = 966). Anti-industry beliefs (indirect effect size = 0.024, 95% confidence interval [CI] = [0.040, 0.056]) and attitudes (indirect effect size = 0.034, 95% CI = [0.006, 0.077]) significantly mediated the effect of the tailored condition on quit intentions. These findings suggest that LGBTQ + -tailored cues in anti-smoking messaging may promote quit intentions indirectly through influencing young adult SMW’s beliefs and attitudes about the tobacco industry. Future campaigns to promote quitting among young adult SMW who smoke should consider incorporating themes to change their beliefs and attitudes about the tobacco industry.

Trial registration This study was registered in ClinicalTrials.gov (NCT04812795) on 24/03/2021.

**Supplementary Information:**

The online version contains supplementary material available at 10.1038/s41598-024-78207-7.

## Introduction

Disparities in cigarette use persist among young adult sexual minority women (SWM), which includes cis- and trans-gender women who have a sexual orientation identity other than heterosexual, in the United States, with young adult SMW having up to 4.8 times greater odds of smoking than heterosexual women^1^. Targeted tobacco marketing^2,3^, minority stressors^4,5^, social norms among the LGBTQ + community^5,6^, and alcohol and drug use^6,7,8^increase the risk of smoking among young adult SMW. As a result, young adult SMW are at significantly higher risk of smoking-related illnesses, including cancer and heart disease^1,9^. Feeling connected to the LGBTQ + community and having peer support are protective factors against smoking among young adult SMW^8^. Smoking prevention and cessation interventions for young adult SMW are necessary to reduce health disparities, but few studies address this need. In 2020, only 1.4% of LGBTQ + health studies funded by the National Institutes of Health (NIH) focused on tobacco use, and 1% of NIH-funded tobacco and health studies focused on LGBTQ + populations^10^.

### Culturally tailored anti-smoking campaigns

Anti-smoking campaigns tailored to the LGBTQ + community are a promising strategy for reducing smoking disparities. Culturally tailored interventions are defined as those that use LGBTQ+-relevant imagery, symbols, and language to engage LGBTQ + audiences^11^. Anti-smoking interventions that are culturally tailored are perceived as more acceptable and promising among LGBTQ + young adults^12^. Sexual minority young adults prefer resilience-focused interventions that are supportive, motivating, and empowering^13,14^, as well as messages that leverage the strengths of the LGBTQ + community^15^.

There is mixed evidence for the effectiveness of culturally tailored tobacco cessation interventions for the LGBTQ + community^16,17^. Some culturally tailored individual and group-based smoking cessation interventions have effectively increased short-term smoking cessation^18,19^. Evidence on the effectiveness of LGBTQ + culturally tailored messaging interventions and campaigns to promote smoking cessation is limited, particularly for Black and Latine LGBTQ + youth and young adults^20^. However, the potential for culturally tailored campaigns to change behavior is promising, as evidence shows that they can effectively influence proximal determinants of behavior. LGBTQ + young adults who had higher positive feelings towards the LGBTQ + community, centrality of their LGBTQ + identity, and belonging to the LGBTQ + community perceived messages from the national LGBTQ+-targeted anti-smoking campaign *This Free Life*to be more effective^21^. *This Free Life*also changed some tobacco-related beliefs^22^.

In a previously published analysis of our experiment that examined the effects of cultural tailoring for both LGBTQ + young adults who smoke and those who do not, we found that culturally tailored campaigns employing counter-industry messaging and messages about the negative health effects of smoking were not more effective for decreasing quit intentions or intentions to purchase cigarettes^23^. However, the tailored campaign messages were associated with a significantly greater increase in anti-industry beliefs and a marginally significant increase in anti-industry attitudes among those who smoke. Previous anti-tobacco campaigns using a counter-industry messaging strategy have effectively decreased tobacco use by promoting anti-industry beliefs and attitudes. We therefore examined in the current analysis whether anti-industry beliefs and attitudes mediated the effect of cultural tailoring on quit intentions.

### Counter-industry messaging mechanisms

Anti-smoking campaigns have frequently employed counter-industry messaging to reduce receptivity to industry messaging and encourage adolescents to be smoke-free^24^. This messaging strategy is based on inoculation theory, which posits that exposing individuals to counterarguments against threatening messages can decrease receptivity to future harmful persuasive messaging^25,26^. The national truth^®^campaign in the US, for example, exposed the tobacco industry’s deceptive marketing practices to increase anti-industry sentiment, decrease receptivity to tobacco industry advertising, and reduce smoking among youth aged 12 to 24^27^. The campaign resulted in greater anti-industry beliefs and attitudes^28,29^, greater mistrust of tobacco companies^24,30^, lower smoking intentions^30,31,32,33,34^, and increased smoking cessation^35^. Consistent with the truth^®^campaign’s theory of effects, anti-industry attitudes and beliefs mediated the relationship between campaign exposure and smoking status^27^. Similarly, statewide counter-industry campaigns have decreased adolescent smoking rates through anti-industry beliefs and attitudes^36,37^.

No studies to date have examined whether anti-industry attitudes and beliefs mediate effects between counter-industry message exposure and quit intentions. Beliefs, defined in this context as the perceived probability that the tobacco industry has a certain attribute (e.g., the industry is targeting vulnerable populations), and attitudes, defined as the “degree of favorableness or unfavorableness” (p. 76) towards the tobacco industry, can influence intentions as proposed by the Reasoned Action Approach^38^. Anti-industry beliefs and attitudes may influence quit intentions, which in turn predict smoking cessation^39^.

Several national anti-smoking campaigns similar to the truth^®^campaign have been implemented to reduce smoking among LGBTQ + people, such as the Truth Initiative and Tips from Former Smokers^40^. Although there is limited research on the effects of these campaigns on smoking behavior, evidence suggests that culturally tailored anti-industry campaigns may be more effective in changing anti-industry beliefs and attitudes among LGBTQ + populations. Skurka et al^41^. found that SGM-targeted and Black-targeted counter-industry ads induced greater anger at the tobacco industry than non-targeted ads among SGM young adults (~ 24% who also identified as Black). Wheldon et al^42^. found that exposure to LGBTQ+-targeted counter-industry ads increased anti-industry beliefs about the industry’s targeting of LGBTQ + people and tobacco use as an LGBTQ + issue. However, these studies did not examine quitting-related outcomes.

Given that culturally tailored anti-smoking campaigns including anti-industry messaging are more effective than non-tailored messages in increasing anti-industry beliefs and attitudes and that anti-industry beliefs and attitudes have mediated the effect of anti-smoking campaigns on smoking status in previous campaigns, we hypothesized that anti-smoking messages tailored for the LGBTQ + community would increase quit intentions through increased anti-industry beliefs and attitudes among the LGBTQ + community. To test this hypothesis, we conducted a randomized controlled experiment to test the effects of tailored compared to non-tailored anti-smoking messages on quit intentions among young adult SMW who smoke.

## Results

### Demographics

Table 1 summarizes participant demographics by experimental condition. Among the overall sample of 966 young adult SMW who currently smoke, 69.5% were aged 24 to 30, and 30.5% were aged 18 to 23. A majority (72.0%) of participants identified as lesbian or gay, 15.3% identified as bisexual, and 12.6% identified as another sexual orientation. Participants most frequently identified as non-Hispanic white (32.8%), followed by non-Hispanic Middle Eastern, Arab, or Arab American (22.7%), non-Hispanic and other racial identity (17.5%), non-Hispanic Black (11.5%), Hispanic (7.5%), and non-Hispanic Asian or Pacific Islander (8.1%). Participants had diverse educational backgrounds, with a majority attaining a four-year college degree or higher (52.1%) and minorities attaining a high school degree or less (23.7%) or some college (24.2%).

**Table 1:** Participant characteristics stratified by experimental condition.

Sample Characteristics	Tailored	Control	Overall
	*N* = 492	*N* = 474	*N* = 966
	*n* (%)	*n* (%)	*n* (%)
Gender Identity^a^			
Cisgender woman	400 (81.3)	400 (84.4)	800 (82.8)
Woman	329 (66.9)	313 (66.0)	642 (66.5)
Transgender woman	13 (2.6)	9 (1.9)	22 (2.3)
Sexual Orientation			
Lesbian or gay	350 (71.1)	346 (73.0)	696 (72.0)
Bisexual	82 (16.7)	66 (13.9)	148 (15.3)
Other	60 (12.2)	62 (13.1)	122 (12.6)
Race^b^			
White	193 (39.2)	189 (39.9)	382 (39.5)
Middle Eastern, Arab, or Arab American	105 (21.3)	114 (24.1)	219 (22.7)
American Indian or Alaska Native	88 (17.9)	70 (14.8)	158 (16.4)
Black or African American	67 (13.6)	65 (13.7)	132 (13.7)
Asian	40 (8.1)	39 (8.2)	79 (8.2)
Native Hawaiian or Other Pacific Islander	2 (0.4)	2 (0.4)	4 (0.4)
Other	5 (1.0)	3 (0.6)	8 (0.8)
Ethnicity			
Hispanic, Latinx, or Spanish	36 (7.3)	36 (7.6)	72 (7.5)
Not Hispanic, Latinx, or Spanish	456 (92.7)	438 (92.4)	894 (92.5)
Age (Years) at Baseline			
18–23	151 (30.7)	144 (30.4)	295 (30.5)
24–30	341 (69.3)	330 (69.6)	671 (69.5)
Education			
High school graduate or less	105 (21.3)	124 (26.2)	229 (23.7)
Some college	125 (25.4)	109 (23.0)	234 (24.2)
College graduate	262 (53.3)	241 (50.8)	503 (52.1)

^a^Participants could select multiple gender identities.

^b^Participants could select multiple races. Two participants in the non-tailored condition did not indicate their race.

*** p < .001; ** p < .01; * p < .05.

### Descriptive analysis and bivariate correlations

Table 2 summarizes descriptive statistics and zero-order correlations of model variables. Table 3 provides descriptive statistics of model variables by experimental condition. There were no significant differences between experimental conditions at baseline or post-test.

**Table 2:** Zero-order Pearson correlation matrix, means, and standard deviations.

	Mean	SD	1	2	3	4	5	6	7
1. Baseline quit intention	3.966	1.346	1.000						
2. Post-test quit intention	5.165	1.513	0.586***	1.000					
3. Baseline anti-industry beliefs	3.572	0.893	0.370***	0.304***	1.000				
4. Post-test anti-industry beliefs	3.842	0.814	0.308***	0.351***	0.624***	1.000			
5. Baseline anti-industry attitude	3.666	0.819	0.316***	0.220***	0.552***	0.429***	1.000		
6. Post-test anti-industry attitude	3.991	0.789	0.268***	0.327***	0.492***	0.520***	0.665***	1.000	
7. Previous quit attempts	4.220	21.698	0.053	-0.030	-0.058	-0.075*	0.014	-0.064*	1.000

*** p < .001; ** p < .01; * p < .05

**Table 3:** Mean (standard deviation) of previous quit attempts, baseline and post-test anti-industry beliefs, baseline and post-test anti-industry attitudes, and baseline and post-test intention to quit cigarettes, by condition.

	Non-Tailored		Tailored		*p*-value
	Mean	(SD)	Mean	(SD)	
Baseline quit intention	3.963	(1.359)	3.968	(1.334)	0.9502
Post-test quit intention	5.176	(1.520)	5.155	(1.508)	0.8321
Baseline anti-industry beliefs	3.585	(0.891)	3.560	(0.895)	0.6643
Post-test anti-industry beliefs	3.807	(0.837)	3.876	(0.790)	0.1907
Baseline anti-industry attitude	3.701	(0.791)	3.631	(0.845)	0.1822
Post-test anti-industry attitude	3.970	(0.780)	4.010	(0.797)	0.4345
Previous quit attempts	4.030	(23.372)	4.404	(19.976)	0.7885

Means were compared using independent t-tests

### Mediation analysis

**Figure. 1:**
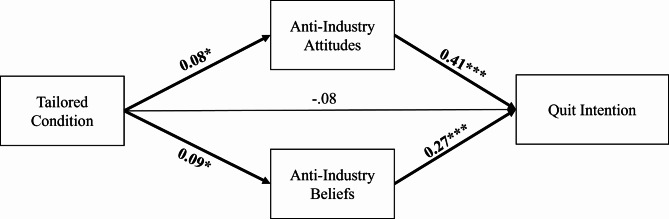
Structural equation model with standardized regression weights. Note: Error correlations and covariates (baseline anti-industry attitude, anti-industry beliefs, quit intention, and quit attempts) not show to reduce visual clutter. * p < .05; ** p < .01; *** p < .001. Model fit: χ2 = 3.196, df = 3, CFI = 1.000, TLI = 0.999, RMSEA = 0.008, SRMR = 0.008.

Figure 1 presents the results of our model, and Table 4 presents the total, direct, and indirect effects estimated using a bootstrapping approach. The direct (effect size [ES]=-0.078, 95% confidence interval [CI] = [-0.215, 0.079]) and total effects (ES=-0.020, 95% CI = [-0.163, 0.148]) of experimental condition on quit intentions were not significant.

**Table 4:** Total, direct, and indirect effects for mediation analyses.

	Observed coefficient	Bias-corrected Bootstrap 95% Confidence Interval	
Total Effects (Direct + Indirect Effects)			
Tailored condition -> Quit intention	-0.020	-0.163	0.148
Direct effects			
Tailored condition -> Quit intention	-0.078	-0.215	0.079
H1: Indirect effects through anti-industry beliefs			
Tailored condition -> Anti-industry beliefs -> Quit intention	**0.024**	**0.004**	**0.056**
H2: Indirect effects through anti-industry attitudes			
Tailored condition -> Anti-industry attitude -> Quit intention	**0.034**	**0.006**	**0.077**

The referent condition is the non-tailored control condition. N = 966. Bolded estimates indicate indirect effects where the bias-corrected 95% CIs did not include zero. Analyses report the effects of tailored condition to quit intention through the specified mediator, adjusting for baseline anti-industry attitude, anti-industry beliefs, quit intention, and quit attempts. The bootstrap procedure was used to compute the bias-corrected 95% confidence intervals with 1000 replications

We hypothesized that both anti-industry beliefs and anti-industry attitudes would mediate the effect of the tailored message condition on quit intentions. The indirect effects of the message condition on quit intention through anti-industry beliefs (ES = 0.024, 95% CI=[0.004, 0.056]) and anti-industry attitudes (ES = 0.034, 95% CI=[0.006, 0.077]) were significant.

In our sensitivity analyses, the fit of the structural equation models in which anti-industry beliefs and attitudes serially mediated the effect of the tailored condition on quit intentions (χ2 = 6.653, df = 6, CFI = 1.000, TLI = 0.999, RMSEA = 0.010; SRMR = 0.009) and post-test quit intention mediated the effect of experimental condition on anti-industry beliefs and attitudes (χ2 = 3.734, df = 3, CFI = 1.000, TLI = 0.997, RMSEA = 0.016; SRMR = 0.008) were similar to that of our hypothesized model.

## Discussion

In this experimental study, anti-industry beliefs and attitudes mediated the effect of messages tailored to the LGBTQ + community on increasing quit intentions among young adult SMW who smoke. These findings align with previous research that found that LGBTQ+-tailored messages increased anti-industry beliefs to a greater extent than non-tailored messages^42^and that youth-targeted counter-industry messaging decreases smoking through anti-industry beliefs and attitudes^27^. Our findings address a key gap in the literature by supporting that anti-smoking ads tailored to young adult SMW are not only more effective in increasing anti-industry beliefs and attitudes but also increase quit intentions through these mediators in this population.

The mediated effect of tailored messaging in our experiment was small, and there was no significant total effect of the tailored condition on quit intentions. The lack of significant total effects may be caused by an unidentified suppressor or a stronger relationship between experimental condition and anti-industry beliefs and attitudes than experimental condition and quit intentions^43^. For example, it is possible that other beliefs, such as those regarding the negative health effects of smoking, mediate the relationship between experimental condition and quit intentions in the opposing direction. Alternatively, a larger dose of tailored messaging may have produced significant direct effects. The small mediation effect found in this study was produced by four brief exposures to tailored messages over the course of four weeks. Tailored messages disseminated in a long-term anti-smoking campaign could lead to larger effects than those observed in this study, including stronger indirect effects and significant direct effects. Even if the effect of tailored messages in a real-world campaign were found to be similar to those in our study, there could be a public health impact if the campaign reached large numbers of people^44^or if the effects accumulated over time^45^.

Notably, anti-industry beliefs and attitudes significantly mediated the effect of tailored messages on quit intentions, even though less than half of the ads shown included counter-industry messaging. The LGBTQ + cues included in the tailored ads may have primed young adult SMW’s connectedness to the LGBTQ + community, which is associated with increased perceived message effectiveness^18^and anti-industry beliefs^42^. We did not examine the effect of the tailored messages on smoking behavior, but prior research has found that anti-smoking campaigns do affect smoking behavior through anti-industry beliefs and attitudes^27^and that quit intentions predict smoking behavior^39^.

In future research, we will investigate whether the tailored messages increase the salience of connectedness to the LGBTQ + community compared to control messages and whether community connectedness mediates the relationship between exposure to tailored anti-smoking ads and anti-industry beliefs. We will also experimentally examine the effects of exposure to both counter-industry and health-related messaging compared to counter-industry or health-related messaging alone to determine which arguments were effective in changing anti-industry beliefs and attitudes. The anti-industry attitudes measure used in this study previously demonstrated high internal consistency among teens and young adults^27,28,36^. However, we found that this measure had poor internal consistency in our sample of young adult SMW. To address this, we omitted one of the three survey items that was not correlated with the other two items. More research is needed to validate anti-industry beliefs and attitudes measures among diverse populations, including young adults in the LGBTQ + community.

Our study has several limitations. Anti-industry beliefs and attitudes were not experimentally manipulated in this study. Therefore, the relationships between anti-industry beliefs and attitudes and quit intentions may not be causal. Beliefs, attitudes, and quit intentions were measured concurrently, so we cannot determine the temporality of these outcomes. Although the prior research suggests that the relationship between the tailored campaign and smoking outcomes is serially mediated by beliefs and attitudes^27^, our model accounted only for the covariance between anti-industry beliefs and attitudes because they were measured concurrently. Although we found that the beliefs, intentions, and attitudes measures demonstrated discriminant validity, we noted that beliefs and attitudes measures at follow-up did not have convergent validity based on the recommended criterion on minimum average variance extracted. Finally, participants were recruited from a non-representative sample of young adult SMW. Our findings may not be generalizable to the U.S. young adult SMW population or to all LGBTQ + populations who use cigarettes.

Although viewing anti-smoking ads containing LGBTQ + cues did not directly influence quit intentions among young adult SMW who smoked cigarettes, we learned that anti-industry beliefs and attitudes may be important intermediate outcomes towards quitting. Changes in these measures following exposure to the ads with LGBTQ + cues were associated with increased quit intentions among young adult SMW. Anti-smoking communication campaigns intended for young adult SMW should consider using LGBTQ + cues in the messaging to promote quit intentions indirectly through influencing their beliefs and attitudes about the tobacco industry.

## Methods

### Sampling and recruitment

The objectives of Project Resist were to co-design a culturally tailored anti-smoking campaign with young adult SMW and to evaluate the effect of tailored messages on smoking and quitting intentions compared to control messages. We recruited a convenience sample of 2,214 young adult sexual minority women in the US, stratified so that approximately half currently smoke (i.e., had at least one cigarette in the past 30 days) between September 2021 and May 2022 to complete a longitudinal online survey. Eligible participants were 18 to 30 years of age and identified as a sexual minority (i.e., a sexual orientation identity other than exclusively heterosexual) and as a cisgender or transgender woman. Participants were recruited through the Prolific online panel, The Population Research in Identity and Disparities for Equality (PRIDE) Study^46^, Instagram ads, ads placed on LGBTQ+-serving community organizations’ social media accounts, and the HER dating app^47^. For this analysis, we excluded people who do not smoke (*n* = 1,002) and those who currently smoke who did not complete follow-up (*n* = 213), completed baseline and follow-up surveys less than 27.5 days apart (*n* = 31), or with missing anti-industry beliefs, anti-industry attitudes, and quit intention items at baseline or follow-up (*n* = 2), resulting in an analytic sample of 966 SMW who currently smoke. The CONSORT flow diagram is presented in Appendix 1.

### Study procedure and data collection

We invited eligible participants to complete five surveys administered using the Qualtrics survey platform: a baseline survey; three booster surveys sent at 1-, 2-, and 3-weeks following baseline; and a follow-up survey sent 4 weeks following baseline. After completing eligibility screening and providing informed consent, eligible participants completed the baseline survey in which they answered questions about their intention to quit, industry beliefs and attitudes, previous quit attempts, and additional participant characteristics. Participants were block randomized, based on sexual orientation and race and ethnicity, using the Qualtrics built-in randomizer function to either the tailored or control condition and were shown 5 randomly selected ads within their assigned condition. Participants were shown 5 additional ads within their condition at each booster survey for a potential total of 20 unique ad exposures over the study period. The order of ad presentation across and within each timepoint was randomized to minimize order effects. Finally, participants completed the follow-up survey that assessed study outcomes and additional participant characteristics. The study was reviewed and considered exempt by the University of Pennsylvania’s Institutional Review Board (protocol no. 843579) and was performed in accordance with the relevant guidelines and regulations. The study was registered in ClinicalTrials.gov (NCT04812795) on 24/03/2021.

### Stimuli

Anti-smoking ads were developed using mixed-methods formative research among young adult SMW and with input from four advisory board members who lead LGBTQ+-serving community organizations and have expertise in tobacco control, cancer prevention and education, and health promotion. Additional details about message development are described in another article currently under review. Message themes included the harms of smoking, benefits of quitting smoking, tobacco industry targeting, and environmental impacts of the tobacco industry. All messages were designed to be non-stigmatizing of people who smoke, evoke positive emotions (except for anti-industry messages), and affirming of LGBTQ + identity. The messages were paired with images of young adult SMW or message-related concepts to create ads. Twenty ads were developed for people who smoke and 20 for those that do not. Fourteen ads were shown to both groups, and 6 were unique to each group. Ads in the tailored condition included a logo with Pride colors (i.e., red, orange, yellow, green, blue, and purple) and a slogan indicating a campaign focus on LGBTQ + health and wellness; ads in the control condition were identical except the same logo was colored yellow, and the campaign slogan indicated a focus on health and wellness generally. Example ads are shown in Appendix 2. The subtle LGBTQ + cues included in the culturally tailored ads were preferred by participants in pretests over more overt cues. As a manipulation check, participants were asked at post-test to indicate whether LGBT, gay or lesbian, bisexual, and transgender populations came to mind when they saw messages over the past month. Participants in the tailored condition were significantly more likely to report that all groups came to mind than those in the control condition (LGBT: 76.6% vs. 67.7%, χ^2^(1, *N* = 966) = 9.55, *p* < .01; Gay or lesbian: 66.9% vs. 58.0%, χ^2^(1, *N* = 966) = 8.08, *p* < .01; Bisexual: 25.0% vs. 15.0%, χ^2^(1, *N* = 966) = 15.11, *p* < .01; Transgender: 19.5% vs. 10.6%, χ^2^(1, *N* = 966) = 15.12, *p* < .01).

### Measurements

#### Intention to quit smoking

We used a 4-item scale to assess intentions to quit smoking, the primary outcome of the study, at baseline and follow-up^48^. Participants rated the following statements on scales ranging from 1 to 7: (1) I will make an effort to quit smoking in the next 30 days (unlikely to likely); (2) I intend to quit smoking in the next 30 days (strongly disagree to strongly agree); (3) I expect to quit smoking in the next 30 days (definitely false to definitely true); (4) How likely is it that you will quit smoking in the next 30 days (unlikely to likely). All items were averaged to construct a scale of quit intentions (Cronbach’s α = 0.90 [baseline] and 0.91 [post-test]).

#### Anti-industry beliefs

We used a 4-item scale to assess beliefs about the tobacco industry at baseline and follow-up^27^. Participants were asked to indicate their agreement with the following four statements: (1) Cigarette companies lie; (2) Cigarette companies target teens to replace smokers who die; (3) Cigarette companies deny that cigarettes cause cancer and other harmful diseases; (4) Cigarette companies deny that cigarettes are addictive. Response options ranged from 1 (strongly agree) to 5 (strongly disagree). All items were reverse coded so that higher values indicate stronger anti-industry sentiment and averaged to create a scale of anti-industry beliefs (Cronbach’s α = 0.84 [baseline] and 0.79 [post-test]).

#### Anti-industry attitudes

We initially used a 3-item scale to assess attitudes toward the tobacco industry at baseline and follow-up^27^. However, the internal consistency of this scale as measured by Cronbach’s alpha was poor (α = 0.65 [baseline] and 0.51 [post-test]) among SMW who currently smoke. We omitted one item, agreement with the statement ‘I would like to see cigarette companies go out of business,’ that was not correlated with the other two items. The included items asked participants to indicate their agreement with the statement ‘I would not work for a cigarette company’ on a scale of 1 (strongly agree) to 5 (strongly disagree) and to indicate how much they like cigarette companies on a scale of 1 (strongly dislike) to 5 (strongly like). Both items were reverse coded so that higher values indicate stronger anti-industry sentiment and averaged to construct a scale of anti-industry attitudes (Pearson’s correlation = 0.53 [baseline] and 0.48 [post-test]). We performed confirmatory factor analysis to test the assumption that the anti-industry beliefs and attitudes measures assess two separate constructs. For baseline and post-test measures, we constructed a structural equation model with anti-industry beliefs and attitudes as separate latent constructs. Based on Hersey et al.’s^37^ measurement model and modification indices, we allowed the errors of two pairs of anti-industry beliefs items to covary: “Cigarette companies deny that cigarettes cause cancer and other harmful diseases” and “Cigarette companies deny that cigarettes are addictive”, and “Cigarette companies lie” and “Cigarette companies target teens to replace smokers who die.” The two-construct model had excellent fit at baseline (χ^2^ = 9.816, df = 6, CFI = 0.998, TLI = 0.996, RMSEA = 0.026, SRMR = 0.012) and post-test (χ^2^ = 10.146, df = 6, CFI = 0.998, TLI = 0.994, RMSEA = 0.027, SRMR = 0.013).

#### Quit attempts

We used one item to assess previous quit attempts at baseline^49^. Participants entered the number of times they stopped smoking for one day or longer because they were trying to quit smoking cigarettes for good in the past 12 months.

### Data analysis

#### Convergent and discriminant validity

We used the CONDISC command in Stata following the SEM command to fit a measurement model of baseline quit intention, anti-industry beliefs, and anti-industry attitudes, omitting any covariances between items. We repeated the procedure for follow-up quit intention, anti-industry beliefs, and anti-industry attitudes, fitting a separate measurement model. The CONDISC command computes the average variance extracted (AVEs) of each construct and the squared correlations (SC) between constructs (e.g., squared correlation between intention and beliefs, beliefs and attitudes, and intention and attitudes). As recommended by Fornell and Larcker^50^, we used a minimum acceptable value of the average variance extracted (AVE) of > = 0.50 to assess the convergent validity of each construct, and an AVE > 0.50 indicates that more than half of the indicator variance is included in the construct score. To assess discriminant validity, we use the criterion that the AVE of each construct being higher than the squared correlations (SC) between constructs. Table 5 summarizes AVEs and SCs for baseline and follow-up constructs. The results of this analysis indicate that constructs for baseline beliefs, attitudes, intentions, and follow-up intentions demonstrated convergent validity (AVEs > 0.5). However, the follow-up beliefs and attitudes constructs had AVEs just under the < 0.5 cutoff (AVEs of 0.489 and 0.482, respectively), suggesting these constructs had less than optimal convergent validity at follow-up. All measures met the discriminant validity criterion (all AVEs of individual constructs are larger than the SCs between constructs).

**Table 5:** Convergent and discriminant analyses of beliefs, attitudes and intentions measures at baseline and follow-up.

Baseline				Follow-up			
	Beliefs	Attitudes	Intentions		Beliefs	Attitudes	Intentions
Beliefs	*0.565*			Beliefs	*0.489*		
Attitudes	0.473	*0.540*		Attitudes	0.479	*0.482*	
Intentions	0.209	0.164	*0.664*	Intentions	0.233	0.234	*0.723*

The results in the diagonals reflect the average variance extracted (AVE) by items within each construct and the results below the diagonals reflect the squared correlations (SC) between constructs. AVEs and SCs were computed after fitting separate measurement models for baseline quit intention, anti-industry beliefs and anti-industry attitudes and follow-up quit intention, anti-industry beliefs and anti-industry attitudes, omitting covariances between items

We conducted a power analysis using GPower version 3.1^51^ to estimate the minimum sample size required to detect the main effect of condition on our primary outcomes, quit intentions for SMW who currently smoke, and smoking intentions for those who do not. Assuming two-tailed tests with 80% power and α = 0.05, the minimum sample size to detect a small effect was 1,600. We factored in 20% attrition to arrive at a sample size of 2,000 at baseline.

We examined bivariate associations between assigned experimental conditions and outcome variables at baseline and post-test using independent t-tests. We fit a structural equation model using Stata 17.0 to examine how exposure to the tailored message condition affects intentions to quit directly and indirectly through anti-industry beliefs and attitudes. The model controlled for baseline anti-industry beliefs and attitudes, baseline quit intentions, and previous quit attempts. An initial model with the hypothesized paths, paths between each control variable and outcome variable, and no correlated errors had a poor fit. We first trimmed insignificant paths between control variables and outcomes using an iterative process in which one insignificant path was removed at a time. Based on modification indices, we added correlated errors between anti-industry beliefs and attitudes. Our final model had an excellent fit (χ2 = 3.196, df = 3, CFI = 1.000, TLI = 0.999, RMSEA = 0.008, SRMR = 0.008).

To examine our hypotheses, we ran the mediation model using a bootstrapping procedure with 1000 replications to obtain bias-corrected confidence intervals of indirect effects. The bootstrap approach did not assume that indirect effect estimates were normally distributed^52^. We conducted two sensitivity analyses by fitting a structural equation model in which (1) anti-industry beliefs and attitudes serially mediated the effect of the tailored message condition on quit intentions and (2) quit intentions mediated the effect of the tailored message condition on anti-industry beliefs and attitudes. The models included the same control variables as our hypothesized model.

## Electronic supplementary material

Below is the link to the electronic supplementary material.

Supplementary Material 1

## Data Availability

Data are not available in a repository. Because we did not specify in the consent process that the data could be used for secondary data analyses, we are not able to make the data public. A restricted dataset may be requested from Andy Tan (andy.tan@asc.upenn.edu) and should include a plan for its use. Data may be made available to qualified researchers after the main findings are published in a peer-reviewed journal. All data sharing will comply with local, state, and federal laws and regulations and may be subject to appropriate human subjects institutional review board approvals.
