# Supplementary material for: Anti-industry beliefs and attitudes mediate the effect of culturally tailored anti-smoking messages on quit intentions among sexual minority women
Source: Sci Rep. 2024 Nov 15;14:28084. doi: 10.1038/s41598-024-78207-7 (PMC11564828; doi:10.1038/s41598-024-78207-7)
Supplement: Supplementary file 1 — Supplementary Material 1 [file 41598_2024_78207_MOESM1_ESM.docx]

**Anti-industry beliefs and attitudes mediate the effect of culturally tailored anti-smoking messages on quit intentions among young adult sexual minority women who smoke: Evidence from a randomized controlled trial**

**Supplementary Materials**

**Appendix 1**

**Project Resist CONSORT Flow Diagram**

Assessed for eligibility (n=11,004)

## Enrollment

Excluded (n= 8,790)

♦  Not meeting inclusion criteria (n= 1,080)

♦  Declined to participate (n= 351)

♦  Other reasons (n= 4,818)

- Quota Met (n = 2,541)

Randomized individuals who smoke (n=1212)

## **Allocation**

Randomized individuals who did not smoke (n=1002)

Not applicable.

Allocated to tailored condition (n=501)

Not applicable.

Allocated to non-tailored condition (n=501)

Analyzed (n=492)
♦ Excluded 107 who did not complete follow up, 14 who were invited erroneously prior to the 1-month follow-up

Analyzed (n=474)
♦ Excluded 106 who did not complete follow up, 17 who were invited erroneously prior to the 1-month follow-up, and 2 who are missing values

Allocated to tailored condition (n=613)

Allocated to non-tailored control (n=599)

## **Mediation Analysis**

## (*Outcome:* Intention to quit smoking)

**Appendix 2**

**Examples of tailored and non-tailored anti-smoking ads**

| Tailored ad for individuals who currently smoke | Non-tailored control ad for individuals who currently smoke |
| --- | --- |
| 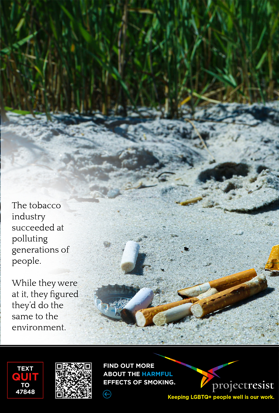 | 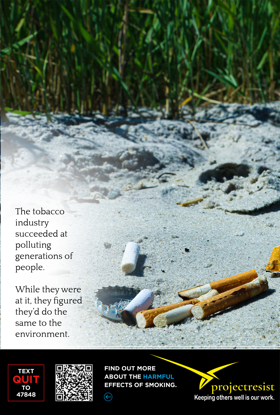 |
